# Functional neuroanatomy of interoceptive processing in children and adolescents: a pilot study

**DOI:** 10.1038/s41598-019-52776-4

**Published:** 2019-11-07

**Authors:** M. Klabunde, H. Juszczak, T. Jordan, J. M. Baker, J. Bruno, V. Carrion, A. L. Reiss

**Affiliations:** 1 Centre for Brain Sciences, Department of Psychology, University of Essex, Wivenhoe Park, United Kingdom; 2 University of California, San Francisco Medical Center, San Francisco, USA; 3 Center for Interdisciplinary Brain Sciences Research, Division of Interdisciplinary Brain Sciences, Department of Psychiatry and Behavioral Sciences, School of Medicine, Stanford University, Stanford, USA; 4 Division of Child and Adolescent Psychiatry, Department of Psychiatry and Behavioral Sciences, School of Medicine, Stanford University, Stanford, USA; 5 Department of Radiology, School of Medicine, Stanford University, Stanford, USA; 6 Department of Pediatrics, School of Medicine, Stanford University, Stanford, USA

**Keywords:** Sensory processing, Human behaviour

## Abstract

In adults, interoception – the sense of the physiological condition of the body - appears to influence emotion processing, cognition, behavior and various somatic and mental health disorders. Adults demonstrate frontal-insula-parietal-anterior cingulate cortex activation during the heartbeat detection task, a common interoceptive measure. Little, however, is known about the functional neuroanatomy underlying interoception in children. The current pilot study examined interoceptive processing in children and adolescents with fMRI while using the heartbeat detection task. Our main findings demonstrate that children as young as the age of six activate the left insula, cuneus, inferior parietal lobule and prefrontal regions. These findings are similar to those in adults when comparing heartbeat and tone detection conditions. Age was associated with increased activation within the dACC, orbital frontal cortex and the mid-inferior frontal gyri. Thus, our pilot study may provide important information about the neurodevelopment of interoceptive processing abilities in children and a task for future interoception neuroimaging studies in children.

## Introduction

Interoception is defined as “the sense of the physiological condition of the body^1^” (p 200). It is a process whereby bottom-up sensory signals from various parts of the body are integrated with top-down cognitive interpretations of these bodily signals^2^. Interoception is important since it facilitates the tracking and regulation of one’s internal state. This directly contributes to one’s sense of self, emotions, empathy, attention, reward processing, and cognitive control^2–9^. Brain regions associated with interoception have been identified as key neural substrates for mental illness^10,11^. Thus, interoceptive dysfunction and its downstream effects may explain numerous symptoms and disorders of psychopathology, making it important for further study in psychiatry^7,12^.

Interoception is often examined using the heartbeat detection task. This task requires participants to indicate each time they feel their heartbeat. Adults activate the frontal-insula-parietal-anterior cingulate cortex (ACC) neural network when performing heartbeat detection during fMRI^13^. Outside the context of heartbeat detection, these regions display dynamic neurodevelopmental changes during childhood and adolescence^14,15^. One study of 1,350 participants demonstrates that children ages six to eleven display intact performance on a behavioral version of the heartbeat detection task^16^. How the brain processes interoceptive stimuli in children and adolescents is unknown. The neurobiological and perceptual changes pertaining to interoception that occur during childhood and adolescence may have important implications for psychopathology development and its treatment.

To examine whether we might be able to study the neurobiology underlying interoceptive processing in children and adolescents, for this pilot study, we modified an fMRI task that is commonly used in adults^17^ and we administered it to children ages six through seventeen years old. This pilot study is the first, of our knowledge, to attempt to examine the neural basis of heartbeat detection in children and adolescents and it was primarily conducted in order to determine whether it is possible to adapt the heartbeat detection task so that it can be used during fMRI to study interoceptive processing in children. Additionally, we hoped to pilot whether: (1) children and adolescents will display similar findings to adults on the heartbeat detection task^13^ and to examine (2) the potential effect of age on activation in brain regions pertaining to interoception in adults.

## Results

### Demographic results

Twelve participants signed assent forms and their parents signed consent forms. One participant was unable to complete the scan due to scanner noise intolerance and was excluded from the study. Eleven participants successfully completed the fMRI study. Participants had a mean age of 11.09 years (SD = 3.56; See Table 1).

**Table 1:** Participant demographics.

Age	Sex	BMI	Baseline HR
6	m	13	90.7
7	m	16	94.2
7	f	15	94
10	f	24	96.9
11	f	20	85.3
11	f	21	71.5
11	m	15	72.8
13	f	21	79.9
13	f	24	74.5
16	m	21	80.11
17	m	28	N/A

### Neuroimaging results

**Figure 1:**
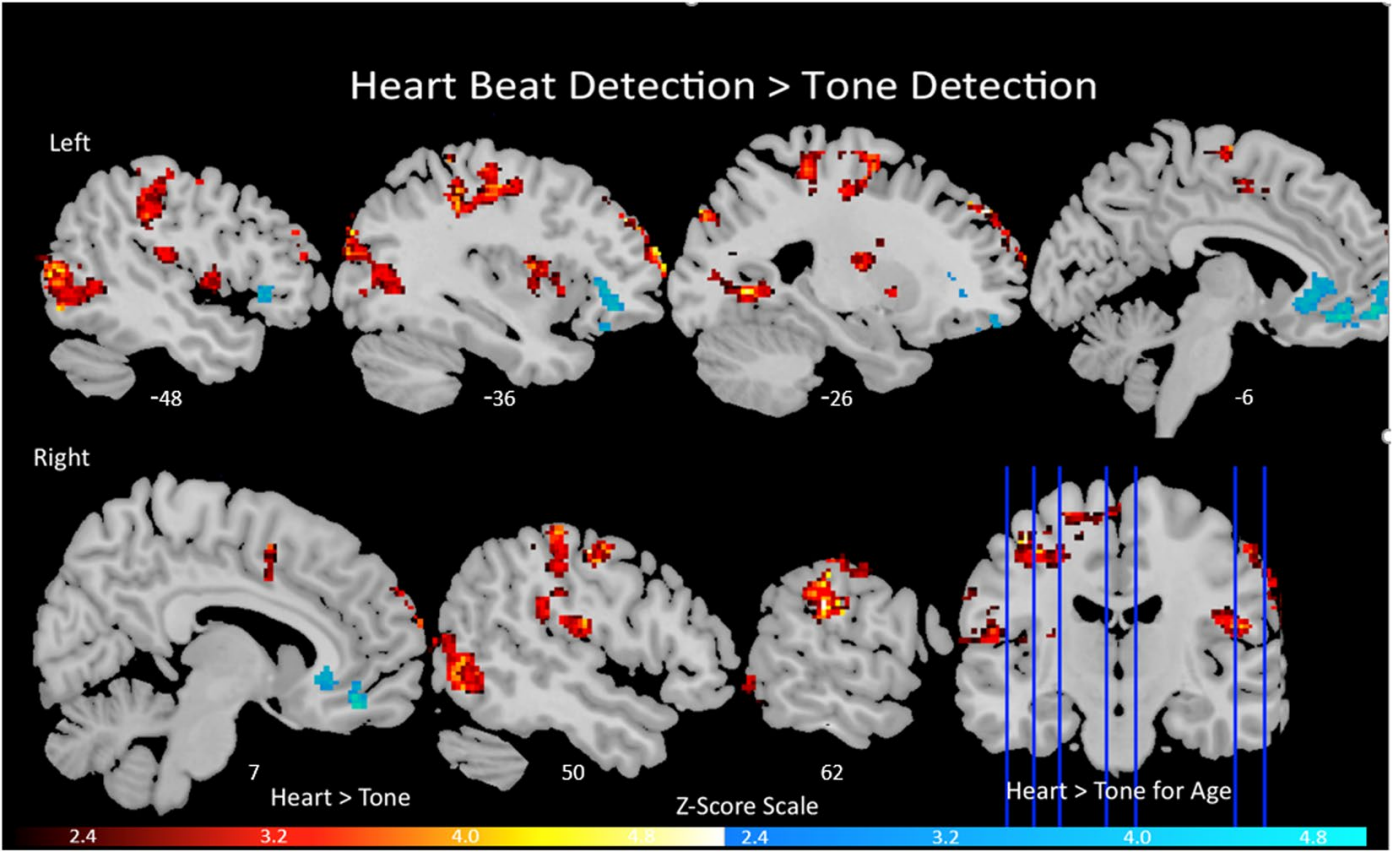
Activation maps obtained from the heartbeat > tone detection contrast. The red demonstrates the regions where activation was significantly greater on the heartbeat condition compared to the tone condition across all participants (*N* = 11) at a cluster correction > 2.3. The blue demonstrates the regions where there was a significant positive relationship between age and greater activation within the heartbeat > tone detection condition.

Increased activity was observed in six clusters (Z = 2.3, family wise error P < 0.05) for the heartbeat > tone contrast. The first cluster encompassed the left post-central gyrus and medial frontal gyrus, the second cluster included the left occipital gyrus, lingual gyrus, middle temporal gyrus, and the cuneus, while the third cluster encompassed the right inferior parietal lobule, precentral gyrus and the postcentral gyrus. Then, the fourth cluster included the left middle frontal gyrus and the superior frontal gyrus, the fifth cluster included the right middle occipital gyrus, middle temporal gyrus, and the inferior temporal gyrus and then, lastly, the sixth cluster included the left insula and the lentiform nucleus of the putamen. Figure 1 shows mean activation during the heart detection > tone detection contrast and Table 2 presents cluster coordinates (in Talairach space) and the corresponding Broadmann Areas within each cluster. No effects for sex or BMI were detected. For the reverse contrast, tone detection > heartbeat detection, no significant activation at a cluster-correction of Z = 2.3 and P < 0.05 was detected for the main analyses or for the age, sex and BMI covariates.

**Table 2:** Brain regions obtained from the heartbeat > tone detection contrasts during group level analyses.

Region	Cluster Size (mm^3^)	Peak Z	Side	BA	Coordinates		
					x	y	z
Postcentral Gyrus	2564	5.61	L	2	−52	−26	46
Medical Frontal Gyrus	—	5.45	L	6	−1	−6	50
Middle Occipital Gyrus	1464	4.95	L	19	−37	−83	14
Lingual Gyrus	—	4.94	L	19	−26	−61	−1
Middle Temporal Gyrus	—	4.87	L	19	−39	−83	17
Cuneus	—	4.63	L	19	−24	−84	32
Inferior Parietal Lobule	1208	5.78	R	40	57	−30	27
Precentral Gyrus	—	5.06	R	4	46	−15	48
Postcentral Gyrus	—	4.93	R	2	53	−29	43
Middle Frontal Gyrus	854	5.91	L	9	−37	39	36
Superior Frontal Gyrus	—	5.71	L	8	−26	44	40
Middle Occipital Gyrus	570	4.62	R	37	38	−62	−1
Middle Temporal Gyrus	—	4.60	R	37	42	−62	4
Inferior Temporal Gyrus	—	4.15	R	37	46	−68	−3
Insula	541	4.21	L	13	−42	−8	4
Putamen- Lentiform Nuculus	—	4.16	L	N/A	−28	5	3

BA = Brodmann Area. R = right; L = left; B = Bilateral; Brain regions were defined by the Talairach atlas. In regions with more than one cluster of activation, coordinates are listed for the cluster with highest activation. Cluster size in mm^3^ and peak activation are listed only for main clusters; activation is not listed for local maxima regions within clusters. The table shows regions where activation was greater in children and adolescents during the heartbeat detection as compared to the tone detection condition at a >2.3 cluster correction.

We detected one significant cluster when examining increased age as a covariate of interest during the heartbeat detection > tone contrast. It included the left medial and middle frontal gyri and the left and right anterior cingulate (ACC). Table 3 presents activation and coordinates for the cluster and Fig. 1 presents BOLD activation during the heartbeat detection > tone detection contrast and BOLD activation during the heartbeat detection > tone detection contrast that is specifically related to the participant’s age.

**Table 3:** Brain regions obtained from the heartbeat > tone detection contrasts while age was included as a covariate during group level analyses.

Region	Cluster Size (mm^3^)	Peak Z	Side	BA	Coordinates		
					x	y	z
Medial Frontal Gyrus	1797	4.97	L	10	−12	54	−1
Anterior Cingulate	—	4.88	R & L	32	12	35	6
Anterior Cingulate	—	4.67	R	24	10	33	2
Middle Frontal Gyrus	—	4.52	L	10	−8	40	−5

BA = Brodmann Area. R = right; L = left; B = Bilateral; Brain regions were defined by the Talairach atlas. In regions with more than one cluster of activation, coordinates are listed for the cluster with highest activation. Cluster size in mm^3^ and peak activation are listed only for main clusters; activation is not listed for local maxima regions within clusters. The table shows regions where activation was related to age and greater in children and adolescents during the heartbeat detection as compared to the tone detection condition at a >2.3 cluster correction.

## Discussion

This pilot study is the first study, to the best of our knowledge, to intentionally examine interoceptive processing with fMRI in children as young as the age of six. Eleven child and adolescent participants completed the heartbeat detection task during fMRI and were subject to fMRI analyses. As hypothesized, we found that it is possible to examine the neural correlates of interoceptive processing in children and adolescents with fMRI as a result of this pilot study. Children and adolescents appear to activate regions similar to adults during a heartbeat (interoceptive) detection task when this task is contrasted to a tone (exteroceptive) detection condition. Such regions include the left insula, left medial prefrontal cortex, and the bilateral inferior parietal lobule^13^. One difference between our findings and those reported in a detailed in meta-analyses of cardioception in adults was the lateralization of insula activation detected in our child participants. Adult findings suggest primary lateralization of insula activation in the right hemisphere, although to this regard the literature reports mixed results^13^. Our results indicate left hemispheric insula activation in children. Additionally, the cuneus (overlapping with the temporal parietal junction) and the inferior parietal lobe were also significantly activated during the heartbeat > tone contrast. These regions are associated with internally directed attention, mentalization, and self-generated thought^18^. Thus, children and adolescents activate regions that are associated with focusing internally during interoceptive tasks.

We also found a positive association between age and BOLD activation during interoceptive processing (heartbeat > tone conditions) within the cingulate cortex (ACC), left and right orbital frontal cortex (OFC), and the left prefrontal cortex (medial and mid-frontal gyrus), suggesting the neurodevelopmental changes occur during interoceptive processing in children. Thus, age related BOLD signal changes may have important implications for the development of interoceptive processing during childhood and adolescence. Developmental theories of interoception suggest distinct stages and critical periods for interoceptive development^19,20^, such as infancy and adolescence. Our preliminary results are consistent with these theories and suggest that interoception is relatively intact in children. However, brain regions consistent with metacognition^21–23^ continue to develop throughout childhood/adolescence^24,25^ and may impact aspects of interoceptive processing. Separating interoception into separate constructs as suggested by Garfinkel *et al*.^26^ may reveal distinct neurodevelopmental trajectories. For example, interoceptive awareness and sensibility may be influenced by the neurodevelopment of brain regions associated with metacognition, such as the ACC and OFC^26^. Additionally, numerous interoceptive signals appear to primarily develop during infancy^27^ and are influenced by oxytocin^28^ and early attachment behaviors^29,30^. Therefore, infancy is also an important period for interoceptive development^28,31^. Such interoceptive signals emphasized in the extant literature include touch, hunger, satiety, and thirst^19,27,32^.

Moreover, identifying distinct developmental trajectories for interoceptive subtypes could help conceptualize and treat psychiatric symptoms. Psychiatric symptoms – such as anxiety and panic symptoms- are characterized by dysfunctional metacognitive processing of interoceptive stimuli and are associated with deficits in interoceptive awareness or sensibility^33^. However, other functional and psychiatric symptoms – such as impaired sensory processing in autism - may be associated with deficits in interoceptive sensory processing. Disorders characterized by interoceptive sensory deficits could be influenced by a genetic predisposition or abnormalities may develop during infancy, making them hard wired. As a result, interoceptive sensory processing deficits may not be immutable to change and could require compensatory strategies -rather than interventions- for the management of its deficits. Interoceptive subtypes associated with metacognitive processing deficits such as disturbances in interoceptive awareness/sensibility, however, may develop during adolescence and alongside the development of the ACC and the prefrontal cortex. Recent studies suggest that a key mechanism of change for cognitive behavioral therapy (CBT) is its influence on metacognition^34^. Therefore, it is possible that these disorders can, therefore, be effectively treated with CBT interventions that correct inaccurate perceptions of one’s internal state.

Interoceptive neurodevelopment may also have important implications for the development of interoceptive prediction. Interoceptive predication suggests that a feedback loop occurs between top-down cortical structures and bottom-up sensory signals. An “error term” is produced, which describes whether an interoceptive signal is or is not congruent with predicted states^2,35,36^. Since regions involved in top-down influences on interoceptive signals appear to develop rapidly during childhood and throughout puberty, these influences may lead to age differences in the weighting of afferent signals during the interoceptive prediction process. Thus, future studies should investigate whether interoception predictive coding theories consistently apply to all age groups and whether/how they may be impacted by developmental variations.

As previously mentioned, study limitations include our pilot study’s small sample size and lack of a longitudinal design. We implemented conservative and accurate mixed effects estimation of brain activation during our fMRI analyses (FLAME 1 & 2), however, to account for the small sample of patients. Nonetheless, future studies with larger samples of children and adolescents should be conducted in order to replicate our fMRI results and interoceptive neurodevelopment should be studied longitudinally.

Another study limitation resides with our choice to examine interoception during fMRI in children and adolescents using the heartbeat detection task^37–39^. Studies have highlighted limitations in measuring interoceptive accuracy with the tracking and discrimination versions of the heartbeat detection task. Limitations to the behavioral versions of the heartbeat detection tasks include: (1) concerns about the reliability and validity of accuracy score^37^ (2) concerns that arousal level could impact task performance, (3) a relationship between task performance and BMI and heartrate^38^. Despite limitations associated with the behavioral heartbeat detection task for measuring interoceptive accuracy, these concerns are not applicable to an fMRI version of the task, which probes brain activation during interoceptive processing instead of obtaining comparable accuracy scores. We decided to adapt the heartbeat detection task to assess the neural correlates of interoceptive processing in children since children were likely to successfully complete this task during fMRI, due to their success completing the behavioral task^16^. Additionally, it was used since there are few non-invasive alternatives for measuring interoception, because it is commonly used within the fMRI environment in adults and due to the available adult comparison data. Moreover, we choose to adapt this particular version of the fMRI heartbeat detection task since it allows us to assess the interoceptive and exteroceptive processing separately in addition to their interactions.

Studies in adults attempt to address concerns about participant arousal during the heartbeat detection task by administering bolus infusions of isoproterenol^39^. Isoproterenol, however, is not currently deemed safe to administer to children and there are no standards for dosing rates in child populations, therefore, making this approach impractical for child populations. Since it is not possible to use isoproterenol to probe arousal in children, we attempted to standardize participant arousal by having participants complete structural and shim scans prior to the functional task. Additionally, to balance our heartbeat and tone detection conditions, however, we delivered tones at a rate that matches the participants’ heartrate. Participants were not aware of their resting heart-rates prior to completing this task since this knowledge can influence interoceptive performance^40^. Regarding concerns about the cofounding effects of BMI on heartbeat detection task performance, we included it as a covariate within our analyses and found no significant activation associated with BMI during interoceptive processing. Therefore, BMI may have less of an influence on the neural signal associated with interoceptive processing than its role in impacting interoceptive accuracy scores.

It is also important to note that the heartbeat detection task requires attention directed towards and conscious appraisal of one’s interoceptive state. Therefore, it is not a “pure measure” of interoceptive signal processing. Future studies should examine whether it is possible to assess interoceptive signal processing without tasks requiring conscious reflection as this could provide a clearer distinction between interoceptive signal processing, awareness and prediction. Additionally, it is unknown whether one interoceptive sense is related to other interoceptive abilities in children. Future studies should examine the relationships between multiple interoceptive senses in children and also whether specific interoceptive senses have unique developmental trajectories during childhood and throughout puberty.

This is the first study, to the best of our knowledge, to directly examine the functional neuroanatomy underlying interoceptive processing in typically developing children and adolescents. We found activation within the left insula, cuneus, parietal and prefrontal regions during interoceptive processing and age variations within ACC, OFC and prefrontal regions. Our neuroimaging results are consistent with previous cardiorespiratory findings in adults, and they support previous behavioral findings that children are can successfully complete interoceptive tasks behaviorally and during fMRI. Overall, our pilot study provides a task that may be used in and could inform the development of larger studies that examine interoception in children. By probing interoceptive circuitry in children, these studies have the potential for further contributing to our understanding of the development and treatment of psychopathology.

## Methods

### Recruitment and screening

Eleven participants, ages 6–17 years, were recruited from the San Francisco Bay Area using flyers, online advertisements, email lists, and a database of individuals who participated in prior studies conducted within our lab. A trained research assistant administered phone screens to interested participants and excluded participants if there was a history of any psychiatric disorders, or significant current or past psychiatric concerns. All screens were reviewed and approved by a licensed psychologist prior to study participation. Additionally, after consenting participants, we administered parent and self-report versions of the Behavior Assessment System for Children (BASC^41^) and excluded participants who exhibited t-scores above 59 (one standard deviation) on either the Behavioral Symptoms Index or the Emotional Symptoms Index. We also recorded the participant’s heartrate and body mass index (BMI). All procedures were approved by the Institutional Review Board at Stanford University and all methods were performed in accordance with the relevant guidelines and regulations. Informed assent and consent from all participants and parents, respectively, were also obtained prior to study participation.

### Procedures

#### Scanner procedures

All participants were scanned prior to 9 am and completed a mock scan and task training trials prior to entering the fMRI scan suite. The mock scan was completed in order to familiarize participants with the scanner conditions and to train them on minimizing head movements. After the mock scan, all participants completed a training trial where they demonstrated an understanding of the task instructions and were required to successfully complete a trial task prior to being accompanied by the research staff to the 3 T scan suite. During the scan, a scanner safe button box was used to record the participants’ responses, bone-conductor pads were used to present auditory stimuli, and a scanner-safe pulse-oximeter recorded the participants’ heart rate. Functional stimuli were administered with EPRIME software and were projected onto a mirror that was attached to the fMRI head coil.

#### Structural scan

All participants were scanned at the Stanford Center for Cognitive and Neurobiological Imaging (CNI) and with a GE 3 T MR 750 scanner. We first acquired a T1 structural scan for co-registration of the functional data. The parameters for the T1 scan were: TR = 6.7 ms; TE = 2.9 ms; flip angle = 12°; number time points = 1; number of slices = 180; 1 mm isotropic voxels; FOV = (256, 256); acquisition matrix = (256, 256); phase encode undersample = 0.4; and the slice encode undersample = 1. Participant heartrate was monitored and recorded during the structural scan.

#### FMRI task acquisition

Prior to the functional scans, a higher order shimming protocol was used to correct for B0 heterogeneity and to avoid blurring and signal loss. To compensate for the loss of power resulting from shortening the scan blocks and runs (see below), we implemented a high-resolution eight multiband gradient Echo Planar Imaging (EPI) scanning sequence with whole brain coverage^42^. A reverse gradient scan was completed and allowed us to calibrate the separate multiband acquisitions (64 unmuxed slices, 12.8 cm). The parameters for the fMRI multiband EPI data were: 2 mm isotropic voxels; 212 × 212 mm field of view (FOV); 482 timepoints; TE = 30 ms; TR = 720 ms, Flip angle = 54.0°, acquisition matrix = 106 × 106, phase encode undersample = 1, slice encode undersample = 1.

#### Heartbeat detection functional MRI task

**Figure 2:**
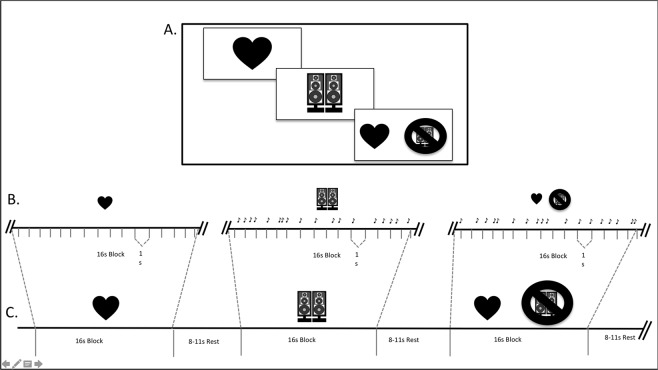
Heartbeat perception functional MRI task for children. Section A displays the visual stimuli that were shown to each participant during each scan condition. Section B presents the approximate pacing of the tones that were presented in the tone and tone inhibition blocks. The rates of tones were individualized to match each participant’s mean heart rate recorded immediately prior to their entry into the scanner. The note indicates the tones that were presented during the scan. They were jittered to generally match the frequency of the standard heart rate. Section C presents a sample time course of each scan run. The heartbeat counting, tone counting and tone inhibition blocks were pseudo-randomized throughout the scan run. Each condition was presented four times and was separated by a jittered inter-stimulus-interval of approximately 10 seconds.

To examine interoception in children during fMRI, we modified a mental-tracking version of the heartbeat detection task that was previously used in adults^17^ by shortening the lengths of the blocks and the length of the scan runs. Each run totaled 330 s and included 16 s blocks of the following three conditions: heartbeat detection, tone detection and a heartbeat counting/tone inhibition block. The three conditions were pseudorandomized and were presented four times per run. A fixation cross separated the blocks and was displayed for an inter-stimulus-interval of 8–11 s (average 10 s; see Fig. 2). In the heartbeat condition, participants were asked to press a button every time they felt their heartbeat. For the tone condition, participants were asked to press a button when they heard a tone over the scanner noise. In the inhibition condition, participants were asked to press a button when they felt their heartbeat and were asked to ignore the tone. To match task difficulty to the heartbeat detection condition, tones were detectable but difficult to hear above the scanner noise. We included the inhibition condition in order to create an optional contrasting condition where participants were required to ignore the tones and focus on detecting their heartbeats since the tone condition required the participants to ignore their heartbeats and focus on detecting tones. After completing the task, the participants reported that it was difficult, but possible, to hear the tones over the scanner noise and detect their heartbeats. The tone rate was individually matched to each participant’s heart rate (ex. 70 bpm), which was not shared with the participants. They were reminded of task instructions immediately prior to the functional task and while lying on the scanner bed. Participants completed four scan runs in order to ensure that usable data was obtained for each participant. We assessed participant compliance after they finished the task. If a participant reported that they were unable to attend to and complete the task during the scan run, the run was excluded from our final analyses and a compliant scan run from that participant was used. We eliminated scans that were too impacted by motion for successful data unwarping since multiband scanning is highly sensitive to the effects of motion. We also examined FSL’s MCFLIRT’s relative motion parameter output metrics (estimated mean displacement) prior to analyses and included scan runs with the lowest relative and absolute motion. Overall, the data presented in this manuscript was successfully unwarped, and included completed runs that demonstrated minimal movement and were reported as “attended to” by the participant. Only one run per participant was included in the group analyses.

### MRI analyses

#### Motion analyses

We regressed out the standard six motion parameters included within the FSL image processing software package^43^ and applied “data scrubbing” procedures as described by Power and colleagues using the FSL motion outliers’ script during preprocessing^44^. During data scrubbing, the union between the root mean squared intensity differences of volume N to volume N + 1 (DVARS; rotation average) and frame displacement (FD; translation parameter differences) were added as additional confound explanatory variables and, thus, were regressed out of our linear model. The cut-off threshold for FD and DVARS was computed automatically using the 75th percentile + 1.5 times the Inter-Quartile Range, as implemented in the fsl_motion_outliers script (supplied with FSL). Additionally, one volume before and two volumes after the union of DVARS and FD were also excluded.

#### FMRI data analysis

Functional imaging data were analyzed and pre-processed using FSL FEAT (FMRI Expert Analysis Tool) Version 6.00. The following preprocessing steps were applied: gradient unwarping of the functional images using the FSL topup tool, motion correction using MCFLIRT, non-brain removal using BET, spatial smoothing using a Gaussian kernel of FWHM 5 mm, grand mean intensity normalization of the entire 4D dataset by a single multiplicative factor, and high-pass temporal filtering (Gaussian-weighted least-squares straight line fitting). Registration to high-resolution structural and standard space images were carried out using FLIRT. Time series statistical analyses were performed using FILM with local autocorrelation correction.

#### Individual subject analyses

Blocks of the heartbeat and the tone detection conditions were convolved with a double gamma hemodynamic response function. Contrasts included heartbeat > tone, tone > heartbeat. The optional inhibition condition was not analyzed and reported in this manuscript. A temporal derivative was used to account for voxel-wise differences in the hemodynamic response and temporal filtering was applied. Voxel wise t-statistic maps for each comparison were generated for each participant.

#### Random effects

Random effects were calculated with FSL’s FLAME 1 + 2. Z-score converted T/F statistic images were thresholded using clusters determined by Z > 2.3 and cluster-corrected significance threshold of *p* = 0.05. For each covariate, which included age, sex and BMI, mean-centering was calculated by obtaining the average value across all of our participants and subtracting this average from each participant’s value. Brain regions corresponding to activation clusters were converted from MNI space to Talairach x, y and z coordinates and subsequently confirmed on the Talairach atlas.

## Data Availability

Data is available by request.

## References

[1] Craig AD (2003). Interoception: the sense of the physiological condition of the body. Curr. Opin. Neurobiol..

[2] Seth AK (2013). Interoceptive inference, emotion, and the embodied self. Trends in Cognitive Sciences.

[3] Craig AD (2009). How do you feel — now? The anterior insula and human awareness. Neuroscience.

[4] Garfinkel SN, Critchley HD (2013). Interoception, emotion and brain: new insights link internal physiology to social behaviour. Commentary on:: ‘Anterior insular cortex mediates bodily sensibility and social anxiety’ by Terasawa *et al*. (2012). Social cognitive and affective neuroscience.

[5] Ernst J, Northoff G, Boker H, Seifritz E, Grimm S (2013). Interoceptive awareness enhances neural activity during empathy. Hum. Brain Mapp..

[6] Farb NA, Segal ZV, Anderson AK (2013). Mindfulness meditation training alters cortical representations of interoceptive attention. Soc. Cogn. Affect. Neurosci..

[7] Khalsa SS, Lapidus RC (2016). Can Interoception Improve the Pragmatic Search for Biomarkers in Psychiatry?. Front. psychiatry.

[8] Paulus MP (2007). Decision-Making Dysfunctions in Psychiatry Altered Homeostatic Processing?. Science (80-)..

[9] Wiens S (2005). Interoception in emotional experience. Curr Opin Neurol.

[10] Craig AD (2002). How do you feel? Interoception: the sense of the physiological condition of the body. Nat. Rev. Neurosci..

[11] Goodkind M (2015). Identification of a common neurobiological substrate for mental Illness. JAMA. Psychiatry.

[12] Khalsa, S. S. *et al*. Interoception and Mental Health: A Roadmap. *Biol. Psychiatry Cogn. Neurosci. Neuroimaging* **3** (2018). 10.1016/j.bpsc.2017.12.004 PMC6054486 29884281

[13] Schulz, S. M. Neural correlates of heart-focused interoception: a functional magnetic resonance imaging meta-analysis. *Philos*. *Trans*. *R*. *Soc*. *B* **371** (2016). 10.1098/rstb.2016.0018 PMC5062106 28080975

[14] Raznahan A (2011). Patterns of coordinated anatomical change in human cortical development: a longitudinal neuroimaging study of maturational coupling. Neuron.

[15] Shaw P (2008). Neurodevelopmental trajectories of the human cerebral cortex. J. Neurosci..

[16] Koch A, Pollatos O (2014). Cardiac sensitivity in children: sex differences and its relationship to parameters of emotional processing. Psychophysiology.

[17] Zaki J, Davis JI, Ochsner KN (2012). Overlapping activity in anterior insula during interoception and emotional experience. Neuroimage.

[18] Benedek M (2016). Brain mechanisms associated with internally directed attention and self-generated thought. Sci. Rep..

[19] Harshaw C (2015). Interoceptive dysfunction: Toward an integrated framework for understanding somatic and affective disturbance in depression. Psychol. Bull..

[20] Murphy J, Brewer R, Catmur C, Bird G (2017). Interoception and psychopathology: A developmental neuroscience perspective. Developmental Cognitive Neuroscience.

[21] Fleming SM, Huijgen J, Dolan RJ (2012). Prefrontal Contributions to Metacognition in Perceptual Decision Making. J. Neurosci..

[22] Fleming, S. M. Relating Introspective Accuracy to Individual Differences in Brain Structure Stephen M. Fleming. Science (80-). **329** (2010). 10.1126/science.1191883 PMC3173849 20847276

[23] Kepecs A, Uchida N, Zariwala HA, Mainen ZF (2008). Neural correlates, computation and behavioural impact of decision confidence. Nature.

[24] Dumontheil I, Burgess PW, Blakemore S-J (2008). Development of rostral prefrontal cortex and cognitive and behavioural disorders. Dev. Med. Child Neurol..

[25] Cachia A (2016). Longitudinal stability of the folding pattern of the anterior cingulate cortex during development. Dev. Cogn. Neurosci..

[26] Garfinkel SN, Seth AK, Barrett AB, Suzuki K, Critchley HD (2015). Knowing your own heart: Distinguishing interoceptive accuracy from interoceptive awareness. Biol. Psychol..

[27] Harshaw C (2008). Alimentary epigenetics: A developmental psychobiological systems view of the perception of hunger, thirst and satiety. Developmental Review.

[28] Quattrocki E, Friston K (2014). Autism, oxytocin and interoception. Neurosci. Biobehav. Rev..

[29] Stern Daniel N. (2018). The Interpersonal World of the Infant.

[30] Fonagy, P. The Social Biofeedback Theory of Affect-Mirroring: The Development of Emotional Self-Awareness and Self-Control in Infancy. In *Affect Regulation, Mentalization, and the Development of the Self*, 10.4324/9780429471643-7 (2018).

[31] Brewer R, Happe F, Cook R, Bird G (2015). Commentary on ‘Autism, oxytocin and interoception’: Alexithymia, not Autism Spectrum Disorders, is the consequence of interoceptive failure. Neurosci. Biobehav. Rev..

[32] Kida T, Shinohara K (2013). Gentle touch activates the prefrontal cortex in infancy: an NIRS study. Neurosci Lett.

[33] Wölk Julian, Sütterlin Stefan, Koch Stefan, Vögele Claus, Schulz Stefan M. (2014). Enhanced cardiac perception predicts impaired performance in the Iowa Gambling Task in patients with panic disorder. Brain and Behavior.

[34] Dobson Keith S. (2013). The Science of CBT: Toward a Metacognitive Model of Change?. Behavior Therapy.

[35] Barrett LF, Simmons WK (2015). Interoceptive predictions in the brain. Nat. Rev. Neurosci..

[36] Seth AK, Suzuki K, Critchley HD (2011). An interoceptive predictive coding model of conscious presence. Front. Psychol..

[37] Brener, J. & Ring, C. Towards a psychophysics of interoceptive processes: the measurement of heartbeat detection. *Philos*. *Trans*. *R*. *Soc*. *B* **371** (2016). 10.1098/rstb.2016.0015 PMC5062103 28080972

[38] Cameron, O. G. *Visceral Sensory Neuroscience: Interoception*. (Oxford University Press, 2002).

[39] Hassanpour Mahlega S, Simmons W Kyle, Feinstein Justin S, Luo Qingfei, Lapidus Rachel C, Bodurka Jerzy, Paulus Martin P, Khalsa Sahib S (2017). The Insular Cortex Dynamically Maps Changes in Cardiorespiratory Interoception. Neuropsychopharmacology.

[40] Murphy J (2018). Knowledge of resting heart rate mediates the relationship between intelligence and the heartbeat counting task. Biol. Psychol..

[41] Merenda PF (1996). BASC: Behavior Assessment System for Children. Meas. Eval. Couns. Dev..

[42] Feinberg DA, Setsompop K (2013). Ultra-fast MRI of the human brain with simultaneous multi-slice imaging. J Magn Reson.

[43] Jenkinson M, Beckmann CF, Behrens TEJ, Woolrich MW, Smith SM (2012). FSL. NeuroImage.

[44] Power JD, Barnes KA, Snyder AZ, Schlaggar BL, Petersen SE (2012). Spurious but systematic correlations in functional connectivity MRI networks arise from subject motion. Neuroimage.

